# Evaluation and diagnosis of pediatric obstructive sleep apnea—An update

**DOI:** 10.3389/frsle.2023.1127784

**Published:** 2023-03-30

**Authors:** Taylor B. Teplitzky, Audrey Zauher, Amal Isaiah

**Affiliations:** ^1^Department of Otorhinolaryngology–Head and Neck Surgery, University of Maryland School of Medicine, Baltimore, MD, United States; ^2^Department of Pediatrics, University of Maryland School of Medicine, Baltimore, MD, United States; ^3^Department of Diagnostic Radiology and Nuclear Medicine, University of Maryland School of Medicine, Baltimore, MD, United States

**Keywords:** sleep apnea, pediatric OSA, PSG (polysomnography), update, pediatric OSA diagnosis

## Abstract

**Purpose:**

Formal overnight polysomnography (PSG) is required to diagnose obstructive sleep apnea (OSA) in children with sleep disordered breathing (SDB). Most clinical guidelines do not recommend home-based tests for pediatric OSA. However, PSG is limited by feasibility, cost, availability, patient discomfort, and resource utilization. Additionally, the role of PSG in evaluating disease impact may need to be revised. There is a strong need for alternative testing that can stratify the need for PSG and improve the time to diagnosis of OSA. This narrative review aims to evaluate and discuss innovative approaches to pediatric SDB diagnosis.

**Findings:**

Methods to evaluate pediatric SDB outside of PSG include validated questionnaires, single-channel recordings, incorporation of telehealth, home sleep apnea testing (HSAT), and predictive biomarkers. Despite the promise, no individual metric has been found suitable to replace standard PSG. In addition, their use in combination to diagnose OSA diagnosis still needs to be defined.

**Summary:**

When combined with adjunct assessments, HSAT advancements may accurately evaluate SDB in children and thus minimize the need for overnight in-laboratory PSG. Further studies are required to confirm diagnostic validity vis-à-vis PSG as a reference standard.

## Introduction

Sleep-disordered breathing (SDB) in children is characterized by disruption of normal respiration and ventilation cycles during sleep (Gipson et al., 2019), and ranges from mild snoring to obstructive sleep apnea (OSA). Pediatric OSA is associated with lower quality of life (Baldassari et al., 2008), behavior and neurocognitive dysfunction (Landau et al., 2012), impaired growth and development (Nieminen et al., 2000), and greater healthcare utilization (Shehan et al., 2023). Risk factors for OSA in childhood include adenotonsillar hypertrophy (Marcus and Loughlin, 1996), obesity (Mitchell and Kelly, 2007), craniofacial anomalies (Cielo and Marcus, 2015), and neuromuscular disorders (Katz, 2009; Marcus et al., 2012). Prevalence estimates for pediatric OSA range from 1.2 to 5.7% (O'Brien et al., 2003; Bixler et al., 2009; Li et al., 2010), although it approaches 60% in obese children (Verhulst et al., 2008). Pediatric OSA is expected to increase in prevalence with rising childhood obesity (Bryan et al., 2021). Adenotonsillectomy (AT) is the first-line treatment (American Thoracic Society, 1996; Marcus et al., 2012; Mitchell et al., 2019), resulting in resolution or improvement in symptoms in most children. In addition to the obese population, several other cohorts are considered high risk for OSA, with management nuanced by complexity. These include children with Trisomy 21, craniofacial anomalies, craniosynostoses, achondroplasia, and with neuromuscular disorders (ElMallah et al., 2017). Though AT is still considered the first line of treatment, these children have higher rates of residual OSA (Rosen, 2011; Zandieh et al., 2013; Cielo and Marcus, 2015; Moraleda-Cibrián et al., 2015; Thottam et al., 2015; Simpson et al., 2018; Zambon et al., 2022), often requiring additional therapy. Positive pressure may be added to treat residual disease or primarily used in those unsuitable for AT (Muntz, 2012; Nehme et al., 2019). Weight loss (Verhulst et al., 2009), alone or supported by a multidisciplinary team (Roche et al., 2020), has also been beneficial. Additional surgical procedures can also be considered, such as hypoglossal nerve stimulation, uvulopalatopharyngoplasty, inferior turbinate reductions, or lingual tonsillectomy (Ravutha Gounden and Chawla, 2022). Treatment in these cases must be tailored to the child, considering their comorbidities.

Types of PSG are described in Table 1. Currently, overnight, in-laboratory level I polysomnography (PSG) is the only approved technology for diagnosis of pediatric OSA (Marcus et al., 2012; Kirk et al., 2017). Level I PSG is attended by a sleep technician in an accredited facility and includes a minimum of seven parameters: electrooculography (EOG), electroencephalography (EEG), chin electromyography (EMG), airflow, respiratory effort, oxygen saturations, and electrocardiography (ECG) (El Shayeb et al., 2014). Pediatric OSA is diagnosed when the PSG reports an obstructive apnea-hypopnea index (AHI), defined as the frequency of partial or complete reduction in air flow per hour, ≥1 (Mitchell et al., 2019). The most common stratification for mild, moderate, or severe disease is based on AHI thresholds of < 5, 5–9, and ≥10 respectively (Marcus et al., 2013).

**Table 1:** Types of polysomnography (Collop et al., 2007).

**Level of PSG**	**Channels**	**Attended?**
Level I	≥7 channels	Yes
Level II	≥7 channels	No
Level III	4–7 channels	No
Level IV	1–2 channels	No

The American Academy of Sleep Medicine (AASM) (Aurora et al., 2011) and the American Academy of Pediatrics (AAP) (Marcus et al., 2012) recommend screening children with SDB with PSG. The American Academy of Otolaryngology—Head and Neck Surgery (AAO–HNS) recommends PSG before AT in children <2 years of age or in those with obesity, craniofacial or neuromuscular disorders, Down syndrome, sickle cell disease, or mucopolysaccharidoses (Mitchell et al., 2019). The AAO–HNS also recommends PSG if the need for surgery is uncertain or if the physical exam does not explain the severity of SDB (Mitchell et al., 2019).

Despite these recommendations, only 10% of children scheduled for AT undergo a PSG (Mitchell et al., 2006). Several barriers exist for level I PSG, e.g., access to a certified sleep laboratory and the limited technical expertise required to diagnose infants and younger children with OSA (Bertoni and Isaiah, 2019). Additionally, the test burden poses unique challenges for children and their families to sleep in an unfamiliar environment while wearing monitoring equipment (Bertoni and Isaiah, 2019). Caregivers must be present during the PSG, which can impact caring for other family members overnight and their productivity the following day. Polysomnography is also expensive, ranging from $1,000 to 4,000 dollars (Bertoni and Isaiah, 2020; Mitchell and Werkhaven, 2020). These issues translate into social and economic burdens for this vulnerable population.

Due to these obstacles, there is a strong need for alternative testing that can approximate PSG results. To date, validated questionnaires (Ahmed et al., 2018; Isaiah et al., 2020; Patel et al., 2020; Wu et al., 2020), single-channel recordings (Kirk et al., 2003; Saito et al., 2007; Álvarez et al., 2017; Hornero et al., 2017; Bertoni et al., 2020), incorporation of telehealth (Paruthi, 2020; Schutte-Rodin, 2020; Castner and D'Andrea, 2022; Griffiths et al., 2022), home-based PSG (Brockmann et al., 2013; Marcus et al., 2014; Ioan et al., 2020; Gao et al., 2021), and biomarkers (Patacchioli et al., 2014; De Luca Canto et al., 2015; Kheirandish-Gozal et al., 2015; Bhattacharjee et al., 2016; Elsharkawi et al., 2017; Teplitzky et al., 2019; Martín-Montero et al., 2022) have been assessed. In this review, we aim to examine innovative approaches to diagnosing pediatric OSA, including exploring combinations of existing technology with the potential for the accurate evaluation of pediatric OSA, thus stratifying the need for traditional overnight PSG.

## Methods

A narrative review of the relevant literature was performed. Sources were identified through PubMed and Google Scholar searches from September 1, 2022, through January 26, 2023. Search terms were broad and included “pediatric OSA,” “pediatric obstructive sleep apnea,” “pediatric home sleep apnea test,” “sleep questionnaires,” “pediatric sleep questionnaires,” “pediatric OSA diagnosis,” “OSA diagnosis,” “videotaping OSA,” “telehealth pediatric OSA,” “HSAT,” “home diagnosis OSA.” Each article was screened for relevance and quality by the authors and included in the review when deemed appropriate.

## Additional approaches

### Questionnaires

The OSA-18 quality of life survey includes 18 symptom-specific questions grouped into five categories: sleep disturbance, physical suffering, emotional distress, daytime problems, and caregiver concerns (Franco et al., 2000). Symptom severity is ranked on an ordinal Likert scale from 1 = none to 7 = all the time. In validation studies of the OSA-18, a total symptom score (TSS) between 60 and 80 correlated with a moderate impact on health-related quality of life. In contrast, a score above 80 indicated a significant impact (Constantin et al., 2010). Ishman et al. (2015) compared OSA-18 responses to PSG metrics. In White children, the specificity and positive predictive value (PPV) of diagnosing OSA using the OSA-18 were 100% when a TSS cut-off of ≥60 and obstructive AHI > 1 were used (Ishman et al., 2015). However, in non-White children, the specificity of these cut-offs was only 67%, with a PPV of 94% (Ishman et al., 2015). The authors also noted a low sensitivity and negative predictive value in all children, indicating an inability to rule out OSA (Ishman et al., 2015). The study concluded that while the OSA-18 questionnaire remains a validated tool to assess the impact of OSA on quality of life, it cannot be used for its diagnosis (Ishman et al., 2015). Constantin et al. (2010) reported that OSA-18 did not accurately diagnose OSA compared to PSG, especially in children with moderate to severe disease. A meta-analysis importantly identified that the OSA-18 could be used as a screening tool for pediatric OSA, though it should not replace PSG for diagnosis (Wu et al., 2020).

The Pediatric Sleep Questionnaire (PSQ) is a validated survey that asks caregivers about the frequency and quality of snoring, breathing problems, mouth breathing, daytime sleepiness, inattention/hyperactivity, and other symptoms (Chervin et al., 2000). Answers to the questions are in a “yes/no/don't know” format (Chervin et al., 2000). Canto et al. (2014) performed a systematic review and meta-analysis of questionnaires to assess for pediatric SDB. The authors noted that the PSQ had sufficient accuracy in screening children for OSA, but it was insufficient to replace PSG (Canto et al., 2014). However, Wu et al. (2020) noted that the PSQ is a sensitive tool for detecting pediatric OSA. The authors also reported that it might be used as a screening tool for OSA. They commented that it could be considered in combination with pulse oximetry in children as an early detection tool (Wu et al., 2020).

Rosen et al. (2015) demonstrated that PSQ symptom scores related to behavior impairment, quality of life, and sleepiness could predict improvement after adenotonsillectomy. Though it could not act as a surrogate for PSG, the authors endorsed its utility as an adjunct to help expect treatment response (Rosen et al., 2015). Patel et al. (2020) studied the predictive accuracy of questionnaires. The sleep-related breathing disorder (SRBD) scale of the PSQ had a sensitivity of 71–84% but a low specificity of 13% (Patel et al., 2020). Also, the area under the receiver operating characteristic (ROC) curve was small, demonstrating poor overall diagnostic accuracy (Patel et al., 2020). They also emphasized the critical role of questionnaires in quantifying the negative impact of SDB on a child's physical and psychological health (Patel et al., 2020). Chervin et al. (2007) demonstrated that the SRBD scale of the PSQ may predict OSA-related neurobehavioral morbidity and response to adenotonsillectomy as well as, and sometimes better than, standard PSG testing. The role of the PSQ may therefore be in screening, assessing SDB-related quality of life, and measuring surgical outcomes in children diagnosed with OSA.

The Children's Sleep Habits Questionnaire (CSHQ) (Owens et al., 2000), is a 45-item questionnaire and includes assessments of parent- and child-reported symptoms among school-aged children aged 4–10 years. Categories of symptoms include bedtime resistance, sleep onset delay, sleep duration, sleep anxiety, night wakening, parasomnias, sleep disordered breathing, and daytime sleepiness (Owens et al., 2000). Items are ranked on a 3-point scale: “usually” if the sleep behavior occurs 5–7 times per week, “sometimes” if it happens 2–4 times/week, and “rarely” for 0–1 times/week. Owens et al. (2000) reported the validity of the CSHQ, concluding it can be used to screen and identify children with sleep disturbances who warrant further testing, though it cannot replace a diagnostic PSG. A summary of validated questionnaires is found in Table 2.

**Table 2:** Validated sleep questionnaires.

**Questionnaire**	**Items**	**Answer format**
OSA-18 Quality of Life (OSA-18)	18 symptom specific questions, 5 categories • Sleep disturbance • Physical suffering • Emotional distress • Daytime problems • Caregiver concerns	Likert scale 1 (none) through 7 (all of the time)
Pediatric Sleep Questionnaire (PSQ)	22 questions asked in the following domains: • Snoring—frequency and quality • Breathing problems • Mouth breathing • Daytime sleepiness • Inattention/hyperactivity • Other symptoms	Yes, no, don't know
Children's Sleep Habits Questionnaire (CSHQ)	45-item questionnaire to assess children age 4–10 years. Categories: • Bedtime resistance • Sleep onset delay • Sleep duration • Sleep anxiety • Night wakenings • Parasomnias • Sleep disordered breathing • Daytime sleepiness	3-point scale: • Usually—symptom is present 5–7×/week • Sometimes—symptom is present 2–4×/week • Rarely—symptom occurs 0–1/× week

Isaiah et al. (2020) used feature selection algorithms to identify main SDB-related symptoms to predict OSA severity. The authors noted that the original questionnaires were not successful at predicting OSA (Isaiah et al., 2020). However, the selected features eliminated redundancy, resulting in improved prediction performance for OSA severity with a high pre-test probability (Isaiah et al., 2020). Follow-up validation replicated the findings (Kennedy et al., 2022), an essential advance in evaluation for pediatric OSA. This new finding does require further assessment, with multi-institutional validation. However, it holds promise as a reasonable alternative to PSG in resource-limited situations.

### Single channel recordings

The level I polysomnogram measures nine parameters (Bertoni and Isaiah, 2019), of which have been studied in isolation to approximate the overall PSG findings. They are grouped by the system learned; sleep, cardiovascular, oximetry, position, effort, and respiratory (SCOPER) (Collop et al., 2011; Bertoni and Isaiah, 2019, 2020). Of these, cardiovascular, oximetry, position, and respiratory can be measured *via* single-channel recordings.

#### Cardiovascular

Overnight electrocardiogram (ECG) recordings have been evaluated as a potential single-channel recording for pediatric OSA diagnosis. Shouldice et al. (2004) investigated the ability to detect OSA in children based on their ECG recordings during overnight PSG. In this study, obstructive apnea was defined as the absence of airflow with continued chest wall and abdominal movement for ≥2 breaths. Hypopneas were defined as 50% or more decrease in nasal airflow with an associated ≥4% desaturation, an arousal, or both but were only scored if the duration was ≥2 breaths. OSA was diagnosed when the obstructive AHI was ≥1. The authors used a different cutoff system for disease severity, with mild having AHI < 10, moderate AHI 10–15, and severe AHI > 15. The authors created a modified quadratic discriminant analysis classification system, which had a sensitivity of 85% and specificity of 81% to diagnose OSA (Shouldice et al., 2004). However, of the four false negatives identified, three were in cases classified as “mild” OSA, indicating slightly reduced accuracy of this system in children with AHI < 10 (Shouldice et al., 2004). Heart rate variability is an ECG-derived measure that may stratify chronic upper airway obstruction due to the detrimental impact of intermittent hypoxia on autonomic control of the heart (Narkiewicz et al., 1998; Baharav et al., 1999; Teplitzky et al., 2019; Bertoni and Isaiah, 2020). Other studies found predictable changes in heart rate variability in confirmed cases of pediatric OSA (Baharav et al., 1999; Nisbet et al., 2013). Avenues for future research include the integration of cardiac rhythm and other screening methods such as questionnaires or other single channel recordings.

#### Oximetry

Brouillette et al. (2000) evaluated pulse oximetry's utility for diagnosing pediatric OSA. In this study, when a child was suspected of having OSA, a positive nocturnal oximetry trend had at least 97% PPV of OSA (Brouillette et al., 2000). The authors reported that oximetry could be used for definitive diagnosis in children with SDB and adenotonsillar hypertrophy (Brouillette et al., 2000). Garde et al. (2019) evaluated pulse oximetry as an OSA screen for different AHI thresholds. The authors found that with AHI cut-offs of 1, 5, and 10, the models showed good accuracy, sensitivity, and specificity (Garde et al., 2019). They argue that pulse-oximetry based OSA screening at different AHI cut-offs defined referral thresholds for in-laboratory PSG (Garde et al., 2019). Wu et al. (2020) performed a meta-analysis and determined that the combined use of the PSQ with pulse oximetry can detect OSA in children, though only if PSG is not available. The authors noted that pulse oximetry had a high specificity for screening children without mild OSA and the highest overall specificity when compared to PSQ and OSA-18 (Wu et al., 2020).

Nixon et al. (2004) developed an overnight oximetry data scoring called the McGill Oximetry Score (MOS), which ranges from 1 to 4. A score of 1 indicates a normal/inconclusive OSA study, and additional evaluation for OSA is required (Nixon et al., 2004). A score of 2 designates mild OSA, 3 means moderate OSA, and 4 is severe OSA (Nixon et al., 2004). The scoring is based on the number of drops in arterial oxygen percent saturation (SaO2) < 90, < 85, < 80%, and a number of clusters of desaturation events (Nixon et al., 2004). The authors found that overnight oximetry can estimate OSA severity using this scoring system, allowing prioritization of diagnostic testing and treatment for those with severe OSA (Nixon et al., 2004). The utility of the MOS has been varied in the literature. Chuanprasitkul et al. (2021) evaluated PSG results after children had nocturnal oximetry in the setting of adenotonsillar hypertrophy. The authors found a high rate of OSA in children with inconclusive overnight oximetry, defined as MOS category 1 (Chuanprasitkul et al., 2021). The deficiency of pulse oximetry in isolation was corroborated by Kirk et al. (2003) who found portable oxygen monitoring insufficient to identify OSA in healthy children.

Pavone et al. (2013) studied the role of serial overnight pulse oximetry readings vs. a single night and the ability to diagnose OSA in children. This study identified night-to-night consistent nocturnal pulse oximetry had diagnostic accuracy for OSA (Pavone et al., 2013). The authors argue that two nights of nocturnal pulse oximetry generate an accurate MOS score, which supports a diagnosis of OSA when the category ≥ 2 (Pavone et al., 2013). Similarly, Horwood et al. (2014) evaluated a treatment algorithm based on the MOS. The authors recommended the use of MOS when there is a need to stratify children in resource-limited scenarios (Horwood et al., 2014). Pavone et al. (2017) found that an abnormal pulse oximetry reading predicted the need for AT, supporting its use in contexts where PSG is not readily available (Pavone et al., 2017). Saito et al. (2007) similarly found that pulse oximetry can be used to determine indications for AT. More recently, Hoppenbrouwer et al. (2021) evaluated night-to-night pulse oximetry variability in children by one overnight hospital-based PSG and subsequent home oximetry for two consecutive nights. They found that overall, there was no significant variability between the measurements in the hospital vs. in the home setting (Hoppenbrouwer et al., 2021).

Despite the utility of overnight pulse oximetry as a screening method alone, it remains inferior to PSG. In situations where PSG is feasible, pulse oximetry is not recommended as a substitute. With an improved understanding of optimizing the data from home overnight oximetry and its approximation of PSG data, it holds promise for use in the future as part of a home-based test and potentially as an independent diagnostic tool.

#### Position

During standard PSG, body position is monitored with apneas, hypopneas, and changes in respiratory patterns (Bertoni and Isaiah, 2019). The most commonly used device is a wrist-worn actigraph (Bertoni and Isaiah, 2019). Less often, body position is detected *via* hip-worn or in-bed pressure sensors (Bertoni and Isaiah, 2019). Actigraphy helps determine several PSG parameters, including total sleep time (TST), wake after sleep onset (WASO), and sleep efficiency (Bertoni and Isaiah, 2019). There are several actigraphs available, including commercially available wristwatches such as the Fitbit Ultra^®^ (Fitbit, San Francisco, CA) and UP^®^ (Jawbone, San Francisco, CA) (Bertoni and Isaiah, 2019). Sleep-focused actigraphs include the Actiwatch-2^®^ (Phillips Respironics, Amsterdam, The Netherlands) and the Motionlogger^®^ Sleep Watch (Ambulatory Monitoring, Ardsley, NY).

Meltzer et al. (2016) compared the Actiwatch-2^®^ to standard PSG for children with suspected OSA. In this study, the authors found that actigraphy underestimated TST and sleep efficiency, although the sensitivity (0.88) and accuracy (0.84) seemed acceptable (Meltzer et al., 2016). Similar findings were noted when comparing additional brands of actigraphs in children and adolescents (Meltzer et al., 2012), with accurate and sensitive estimations of TST, WASO, and sleep efficiency, though with poor specificity (Toon et al., 2016). The results have been varied, as other studies found commercially available actigraphs poorly approximate sleep compared to PSG (Meltzer et al., 2015). Bertoni et al. (2020) utilized machine learning to generate models which compared overnight PSG parameters to nocturnal actigraphy with the oxygen desaturation index. The goal was to determine if the combination of actigraphy data with oximetry can approximate severe OSA. The authors demonstrated that actigraphy combined with oximetry data could screen for severe OSA (Bertoni et al., 2020), which has implications on postoperative management after AT, as well as a potential diagnostic alternative.

As the data is mixed, further research is needed to help determine the utility of actigraphy in children, particularly in young children who are not yet school age. Given the safety and ease of application, using actigraphy as part of a home sleep evaluation would be beneficial. Their use alone to diagnose OSA is unlikely, though in combination with other single-channel devices shows promise. Further research is needed to assess how to use this technology in the pediatric population effectively.

#### Respiratory

The AASM suggests that respiratory events be captured using an oronasal thermal airflow sensor or a nasal pressure transducer (Bertoni and Isaiah, 2019). These devices are limited mainly to use in the research setting. There have been studies testing single-channel nasal airflow pressure transducers in diagnosing adult OSA, with some devices having better prediction of OSA than others (Rofail et al., 2010a,b). Small studies have been performed in infants and children regarding the utility and accuracy of using a nasal cannula to detect sleep abnormalities. Trang et al. (2002) evaluated 14 infants to assess the ability of a nasal cannula to detect apneas and hypopneas. The authors found that the nasal cannula was better able to detect hypopneas vs. apneas than a thermistor (Trang et al., 2002). However, an observational study comparing nasal cannula pressure to nasal airflow thermistors in detecting apneas and hypopneas identified that the cannula could detect more events than the thermistor (Serebrisky et al., 2002). This more extensive study in 47 children aged 2–14 years is essential, demonstrating a possible single-channel system for identifying sleep disturbances in children (Serebrisky et al., 2002). However, a recent publication based on 172 children under age 3 demonstrated limited ability for the nasal cannula to detect obstructive events (Jurado et al., 2022). Based on these data, the use of current technology for respiratory monitoring in children is limited. However, its use in combination with other testing metrics or surveys has yet to be evaluated.

#### Telehealth

The COVID-19 pandemic increased the scope and reach of telehealth services. Sleep medicine is no exception to this movement, demonstrating increased utilization over the pandemic (Paruthi, 2020). Physicians have developed protocols by which to apply telehealth in the evaluation of children with sleep complaints (Witmans et al., 2008). Some pediatric sleep disorders can be adequately evaluated *via* telehealth appointments, particularly when a physical exam does not alter management. For example, circadian rhythm disorders, insomnia, and sleep-related movement disorders can be managed by telemedicine (Paruthi, 2020). Sleep apnea, however, does require a formal oropharyngeal exam and therefore is not always amenable to telehealth at the initial diagnostic encounter. Though possibly used to screen who requires an in-office exam, telehealth's role in diagnosing pediatric OSA has been challenging. However, incorporating telehealth into HSAT has allowed successful diagnosis in 80% of children around age 10 (Griffiths et al., 2022). This demonstrates an essential caveat to newer technologies, in that study of their use in combination is only just beginning. The use of telehealth as an adjunct during diagnostic testing may prove very beneficial, as demonstrated by Griffiths et al. (2022). Further research is required to assess other benefits of telehealth in pediatric OSA.

#### Videotaping during sleep

The ability of sleep video recordings to approximate PSG findings, or add to home testing, has been studied. Sivan et al. (1996) published the first article describing utility of home video recordings of sleep in screening for OSA. Jacob et al. (1995) similarly noted the value of adding videotape recordings to home testing in children. Lamm et al. (1999) described home videotapes as a useful screening tool in snoring children, though found they cannot distinguish OSA from primary snoring. Given the almost ubiquitous use of smart phones with video technology, Thomas R. J. et al. (2022) recently tested a scoring system for short home sleep videos taken by caregivers during episodes of concerning breathing. The authors found that low scores ruled out moderate-severe OSA, while scores ≥ 3 showed a sensitivity of 100%, specificity of 36%, positive predictive value of 53%, and negative predictive value of 100% for moderate to severe OSA (Thomas R. J. et al., 2022). They concluded that this newly validated clinical scoring system is valuable in triaging children with SDB (Thomas R. J. et al., 2022). Despite successful computer-based analysis of video images in adult OSA (Abad et al., 2016; Muñoz-Ferrer et al., 2019), comparable work is lacking in children. Larger studies are needed to elucidate the role of home videos for the screening and diagnosis of pediatric OSA.

#### Home-based PSG

HSAT aims to replicate the results of an in-hospital PSG with increased comfort and accessibility but lower cost and decreased resource utilization (Kirk et al., 2017; Bertoni and Isaiah, 2020). Typically, HSAT is achieved by combining specific PSG channels *via* portable or wearable sensors (Bertoni and Isaiah, 2020). Once determined to be accessible and feasible (Brockmann et al., 2013; Marcus et al., 2014; Ioan et al., 2020; Lildal et al., 2021), multiple studies have been performed to assess the adequacy of pediatric HSAT.

Gao et al. (2021) performed a systematic review and meta-analysis to determine the diagnostic accuracy of portable, at-home PSG in children. Home testing showed better specificity than sensitivity, indicating appropriate use as a screening method for OSA (Gao et al., 2021). Brunetti et al. (2001) proposed an algorithm to use home testing as a screening tool to help better utilize overnight in-lab testing.

Scalzitti et al. (2017) performed a prospective comparison of home testing to PSG in children aged 2–17. Despite the variability in results, the AHI and lowest oxygen saturation measurements were similar between tests in children aged ≥ 6 years (Scalzitti et al., 2017). These results were echoed by Withers et al. (2022) who compared level I hospital-based PSG to level II home PSG in children aged 5–16. The authors found that level II PSG had an excellent correlation with level I PSG, with the benefit of higher sleep efficiency (Withers et al., 2022). The conclusion of this report was that level II PSG is can be considered for diagnosis in children aged 5–18 (Withers et al., 2022). A study by Alonso-Álvarez et al. (2015) identified home respiratory polygraphy as a potentially helpful and reliable approach for OSA diagnosis in children compared to in-lab PSG. Bhattacharjee et al. (2021) noted acceptable agreement between AHI and oxygen desaturation index between the home and in-laboratory portable monitors, and in-laboratory PSG in 20 adolescents. When installed correctly by trained technicians, home unattended respiratory polygraphic recordings can be used for OSA screening in otherwise healthy children (Ioan et al., 2023). HSAT results may be improved and comparable to in-lab PSG, with the addition of attendance by an online video technician (Green et al., 2022). These results are essential, as they demonstrate a potential population that may be tested at home, creating more availability for children who need PSG for diagnosis. Additionally, the combination of HSAT with screening questionnaires has been beneficial in identifying (Revana et al., 2022), or excluding (Maggio et al., 2021), moderate to severe OSA, further supporting the use of alternate testing methods for improved accuracy.

At this time, the role of HSAT in children is limited to a screening tool, as the AASM does not support the use of HSAT as a replacement for PSG to diagnose OSA in children (Kirk et al., 2017). Difficulty in feasibility, validity, identifying arousals and hypoventilation, issues with use in young children or children with comorbidities, and differences in body sizes are cited as the key limitations (Kirk et al., 2017). The need for home testing is evident, though to date some of the obstacles related to the mechanics of testing have not been overcome. As more studies are performed showing positive outcomes, the guidelines regarding HSAT may eventually evolve.

#### Biomarkers

Untreated OSA has lasting implications on overall health and physiology (Archbold et al., 2012; Marcus et al., 2012; Teplitzky et al., 2019). The study of the systemic impact of OSA has led to evaluation of biomarkers as an alternate means, or adjuncts, for the diagnosis of OSA. From a cardiovascular standpoint, untreated OSA is associated with alterations in left ventricular mass and wall thickness, end diastolic dimensions, and interventricular septal thickness (Amin et al., 2002; Bhattacharjee et al., 2009; Teplitzky et al., 2019). Pediatric OSA can result in right heart failure from pulmonary hypertension, resulting from alveolar hypoventilation from the cyclic apneas, and subsequent pulmonary vasoconstriction (Bhattacharjee et al., 2009; Koc et al., 2012; Teplitzky et al., 2019). For these reasons, pre-operative cardiovascular assessment has been considered, though with debated utility (Li et al., 2008a; Teplitzky et al., 2019; Martín-Montero et al., 2022).

An association between OSA and inflammation was first described by Tauman et al. (2004) who showed that elevated levels of plasma C-reactive protein (CRP), a known marker of inflammation, correlated with AHI, oxygen nadir, and arousal index in some children with OSA (Tauman et al., 2004). This elevation in CRP was also associated with development of cardiovascular disease (Dos Santos et al., 2008), particularly in the setting of obesity (Choi et al., 2013). A large body of evidence has developed evaluating the role of CRP in OSA management. Kheirandish-Gozal et al. (2006) demonstrated elevated CRP levels prior to treatment, with reduction in CRP levels after adenotonsillectomy, adding evidence to support OSA leads to systemic inflammation. This finding has been supported by several authors, noting improvement in systemic inflammation and reduced CRP after OSA treatment (Li et al., 2008b; Ingram and Matthews, 2013; Mutlu et al., 2014; Nachalon et al., 2014; Van Eyck et al., 2014). CRP may also be able to identify residual OSA after adenotonsillectomy (Bhattacharjee et al., 2016). However, the role of CRP in pediatric OSA is nuanced, as other research has noted that CRP in isolation is not predictive of OSA (Kheirandish-Gozal et al., 2015), due to confounding factors such as interindividual variability, environmental, and genetic factors (Kheirandish-Gozal and Gozal, 2017).

Additional metabolic markers have been assessed, given the increasing rates of pediatric obesity (Childhood Obesity Facts, 2022), and concomitant obesity in children with OSA (Bachrach et al., 2022). Comorbid diagnoses to obesity, such as insulin resistance and dyslipidemia, have been evaluated in pediatric OSA (Deboer et al., 2012; Zong et al., 2013; Bhushan et al., 2014; Amini et al., 2017; Siriwat et al., 2020). The use of insulin and lipid levels are not suitable for the diagnosis of OSA, but do denote systemic involvement of the disease, and may serve as an adjunct in global evaluation and work up.

Salivary evaluation has identified OSA biomarkers (Patacchioli et al., 2014; Bencharit et al., 2021), some of which have reportedly acceptable diagnostic accuracy (Canto et al., 2015). Urinary biomarkers have also been studied with mixed results (Biyani et al., 2018). However, some values have the possibility to be used in diagnosis and prediction of OSA severity in children (Villa et al., 2014; Thomas S. et al., 2022). To date, studies on these topics are relatively small in number without reproducibility, limiting their use as a surrogate for PSG. However, their implications on the broad effects of OSA with systemic involvement are apparent. Further studies are needed to better evaluate the role of biomarkers in OSA diagnosis.

#### Special populations

High-risk populations include those with Trisomy 21, craniofacial anomalies, and neuromuscular disorders (ElMallah et al., 2017). Alterations in craniofacial measurements, commonly observed in children with syndromes such as Trisomy 21, have been associated with OSA (Katyal et al., 2013; Sutherland et al., 2020). Consideration of body mass index (BMI) and BMI percentile should be assessed in all children to identify those with obesity. Obesity may cause, or worsen OSA, and increases the risk of persistent OSA after AT (O'Brien et al., 2006; Andersen et al., 2016). Obesity is a common comorbidity in children with Trisomy 21, making their OSA more challenging to manage. Children with Trisomy 21 typically are also hypotonic, perpetuating their airway collapse and OSA (Ali Khan, 2022). A multidisciplinary approach between otolaryngology, plastic surgery, pediatric dentistry, and pulmonology/sleep medicine for management of OSA high risk children can improve postoperative AT outcomes and treatment of persistent OSA (DeVries et al., 2020).

Other special populations include children with cleft palate, in whom the underdevelopment and unusual orientation of the palatal musculature increase the propensity for airway collapse (Robison and Otteson, 2011; Muntz, 2012). Surgical procedures designed to repair these anatomic problems are also associated with a higher risk of postoperative upper airway obstruction, requiring a high degree of vigilance following surgery (Rose et al., 2002; Bergeron et al., 2019).

Results of screening tools for OSA in children with Trisomy 21 have been mixed. Several authors have found use of questionnaires unreliable (Grantham-Hill et al., 2020; Skotko et al., 2023). However, Hill et al. (2018) successfully screened for moderate to severe OSA *via* home pulse oximetry, helping determine which children need formal PSG to confirm OSA. New technologies are emerging to aid in OSA diagnosis (Bassett and Musso, 2017), particularly in these complicated children. Brockmann et al. (2016) studied the feasibility of home PSG in children with Trisomy 21, and obtained a technically successful and acceptable home PSG in 83% of children, concluding that portable home PSG devices may be considered for diagnosis. Ioan et al. (2022) described the utility of pulse transit time (PTT), a technology shown to detect subcortical autonomic arousals, with ventilator polygraphy (PG) to diagnose OSA in children with Trisomy 21. The authors noted a specificity of 1.0 for oAHI >1 event/hour on PTT-PG. When the autonomic arousal index (PTTAI) on PTT-PG is added, the sensitivity for oAHI > 1 is 1.0. The authors concluded that the use of PTT-PG and PTTAI can be diagnostic, though is dependent on signal quality (Ioan et al., 2022). To assess areas of persistent obstruction, cinematic magnetic resonance imaging (“cine MRI”) uses standardized MRI algorithms to localize regions of persistent airway obstruction (Manickam et al., 2016; Isaiah et al., 2018). Another option is drug-induced sleep endoscopy (DISE), with a recent consensus statement on its use in children (Baldassari et al., 2021). In DISE, clinicians can identify potential sites of airway obstruction during sleep using fiberoptic endoscopy to visualize endoluminal upper airway obstruction under anesthesia. The principal uses of cine MRI and DISE are to tailor treatment of upper airway obstruction in children with craniofacial syndromes and potentially in those with recidivism related to OSA. The discussion of OSA in complex children is nuanced and detailed, deserving of its own review. However, it is helpful to address these additional metrics used with PSG, to facilitate personalized approaches for treating pediatric OSA.

## Conclusions

Diagnosis of pediatric sleep disordered breathing is limited by access to level I in-lab PSG. Due to the economic burden of this test, patient-family inconvenience, and limited accessibility, additional methods for diagnosis are needed. Though many avenues have been explored, none in isolation has been satisfactory as a replacement to PSG. Further validation efforts are needed to confirm the adequacy of single channel vs. combined channel recordings in the home setting, though data to date are promising. Similarly, adjunct evaluations such as questionnaires and biomarkers, may prove effective when used in conjunction with some of the SCOPER technologies. Newer studies on pediatric HSAT show promise and may eventually be a reasonable option in certain populations. Additional large volume studies are required, but with the great and persistent need for more accessible diagnostic testing, continued research will hopefully identify acceptable and accurate alternatives to level I PSG.

## Author contributions

TT, AZ, and AI: manuscript drafting and revision and final manuscript approval accountable for all aspects of the work.
